# Hybrid Approach for Correction of Recurrent Aortic Arch Obstruction After Repair of Interrupted Aortic Arch—A Case Report

**DOI:** 10.1016/j.shj.2022.100088

**Published:** 2022-09-14

**Authors:** Maria Nucera, Martin Glöckler, Hannah Widenka, Jan-Oliver Friess, Matthias Siepe, Alexander Kadner

**Affiliations:** a Department of Cardiac Surgery, Center for Congenital Heart Disease, Bern University Hospital, University of Bern, Bern, Switzerland; b Department of Cardiology, Center for Congenital Heart Disease, Bern University Hospital, University of Bern, Bern, Switzerland; c Department of Anaesthesiology and Pain Medicine, Bern University Hospital, University of Bern, Bern, Switzerland

**Keywords:** Congenital heart disease, Hybrid intervention, Interrupted aortic arch, Pediatric, Recurrent aortic arch obstruction

## Background

Recurrent aortic arch obstruction (RAAO) following repair of interrupted aortic arch (IAA) occurs in up to 50% of patients. Treatment options are surgical correction or percutaneous catheter intervention. Balloon angioplasty has become more popular over the last 3 ​decades, but current studies suggest that it may be less effective than surgical treatment.^1^^,^^2^

Consequently, the majority of RAAO requires surgical redo correction involving extensive dissection and deep hypothermic circulatory arrest with a potential higher complication rate.

We report a case of an alternative less invasive hybrid approach in a child with RAAO after IAA correction.

## Case Presentation

We report the case of a 7-year-old child with an IAA type C, an ostium secundum atrial septal defect, and a muscular ventricular septal defect. Aortic arch reconstruction had been performed by using an autologous pericardial patch at day 4 after birth. Following this, the child developed re-stenoses, which were treated with balloon dilatations 4 times.

**Figure 1:**
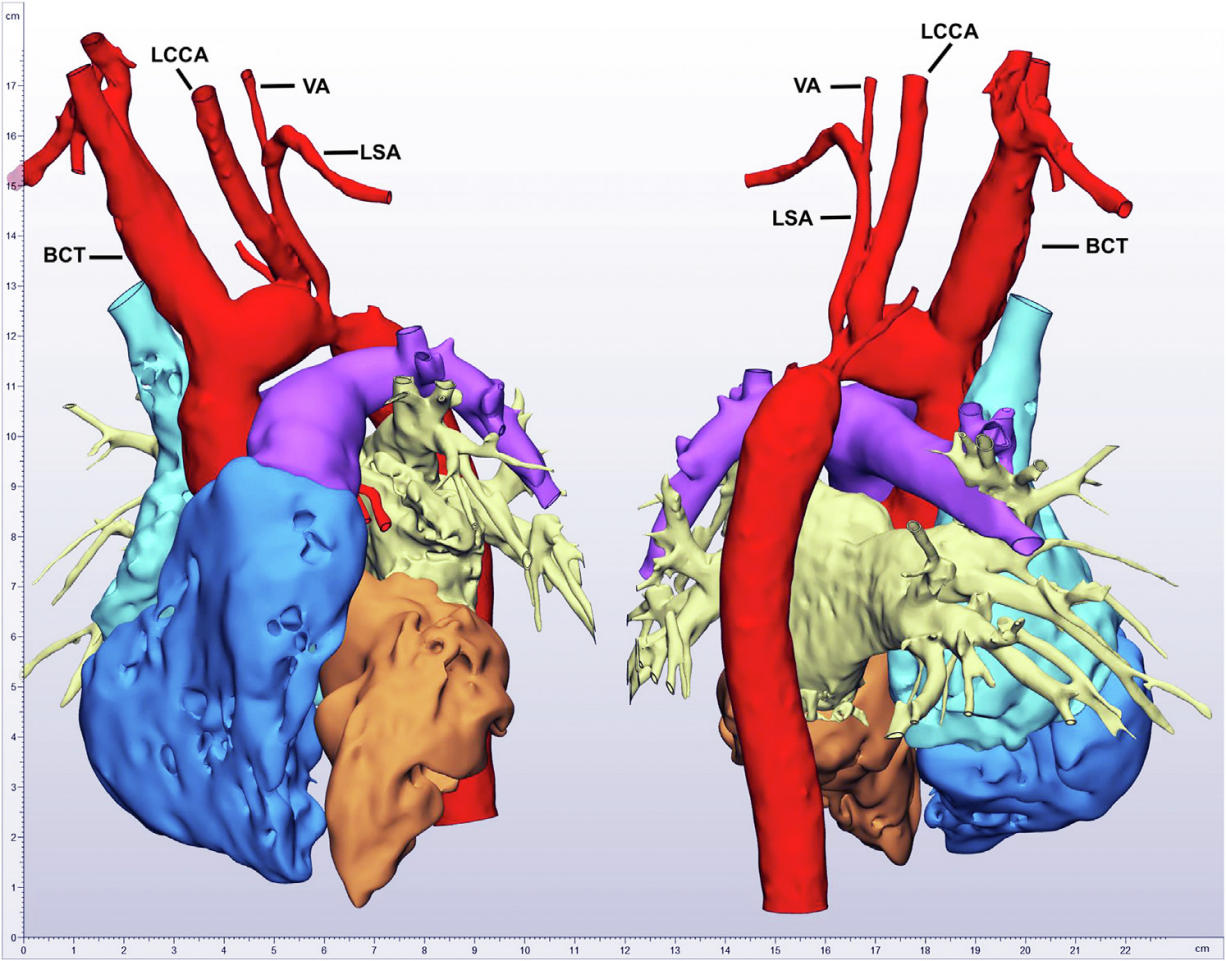
**Three-dimensional-model reconstruction of preoperative computed tomography angiography.** Abbreviations: BCT, brachiocephalic artery; LCCA, left common carotid artery; LSA, left subclavian artery; VA, vertebral artery.

At the age of 7 years, the child presented with a reduced exercise capacity and fatigue. The child was under no medical treatment. A severe re-stenosis (peak systolic gradient 60 mm Hg) of the distal aortic arch involving the offspring of the left common carotid artery (LCCA) was diagnosed (Figure 1).

It was decided to proceed with a hybrid surgical and interventional approach consisting of carotid artery translocation and distal arch stenting. Preoperatively, the patency of the circle of Willis was confirmed, and intraoperatively continuous near-infrared spectroscopy monitoring was performed.

### Hybrid Approach

**Figure 2:**
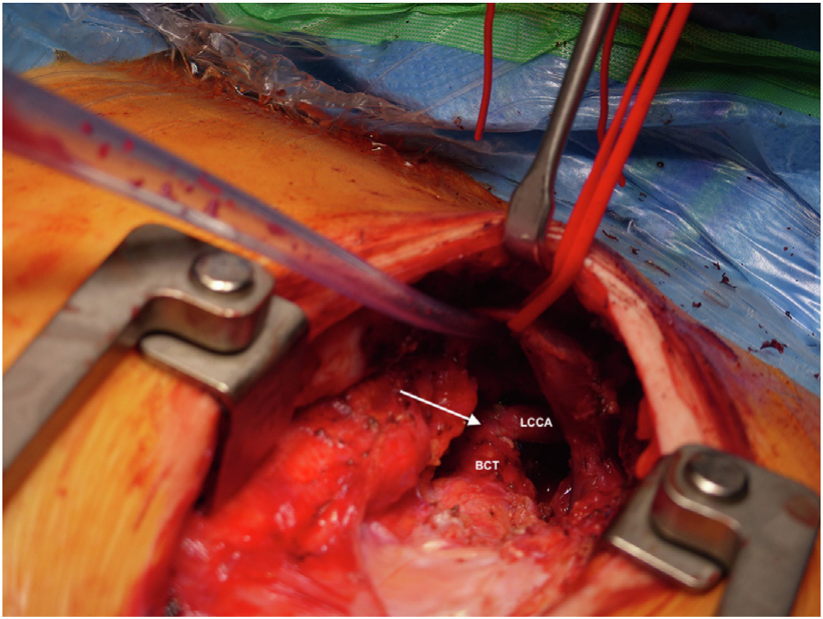
**Intraoperative picture showing carotid artery translocation with an end-to-side anastomosis to the brachiocephalic trunk (arrow).** Abbreviations: BCT, brachiocephalic artery; LCCA, left common carotid artery.

After median re-sternotomy, the ascending aorta was exposed. LCCA and brachiocephalic trunk were dissected. The offspring of LCCA from the aortic arch was visualized, divided, and oversewn. LCCA was liberally mobilized and re-anastomosed in an end-to-side fashion to the partially clamped brachiocephalic trunk (Figure 2).

Access to the right femoral artery was obtained percutaneously with insertion of a 4-Fr sheath. Afterward, the 4-Fr sheath was exchanged with a 10-Fr sheath. The guidewire was advanced in the ascending aorta, and intraoperative aortography was obtained. A IntraStent^TM^ LD Max^TM^ (ev3 Inc. Plymouth, MN, USA) of 26 mm length was advanced and deployed with a 12-mm balloon just distal to the brachiocephalic artery. The outlet of the subclavian artery was covered by this uncovered stent. Invasive blood pressure measurement revealed a residual gradient of 10 mmHg between the right radial and femoral arteries.

### Postoperative Phase and Follow-Up

The patient was extubated immediately after the procedure and had an uneventful postoperative course.

**Figure 3:**
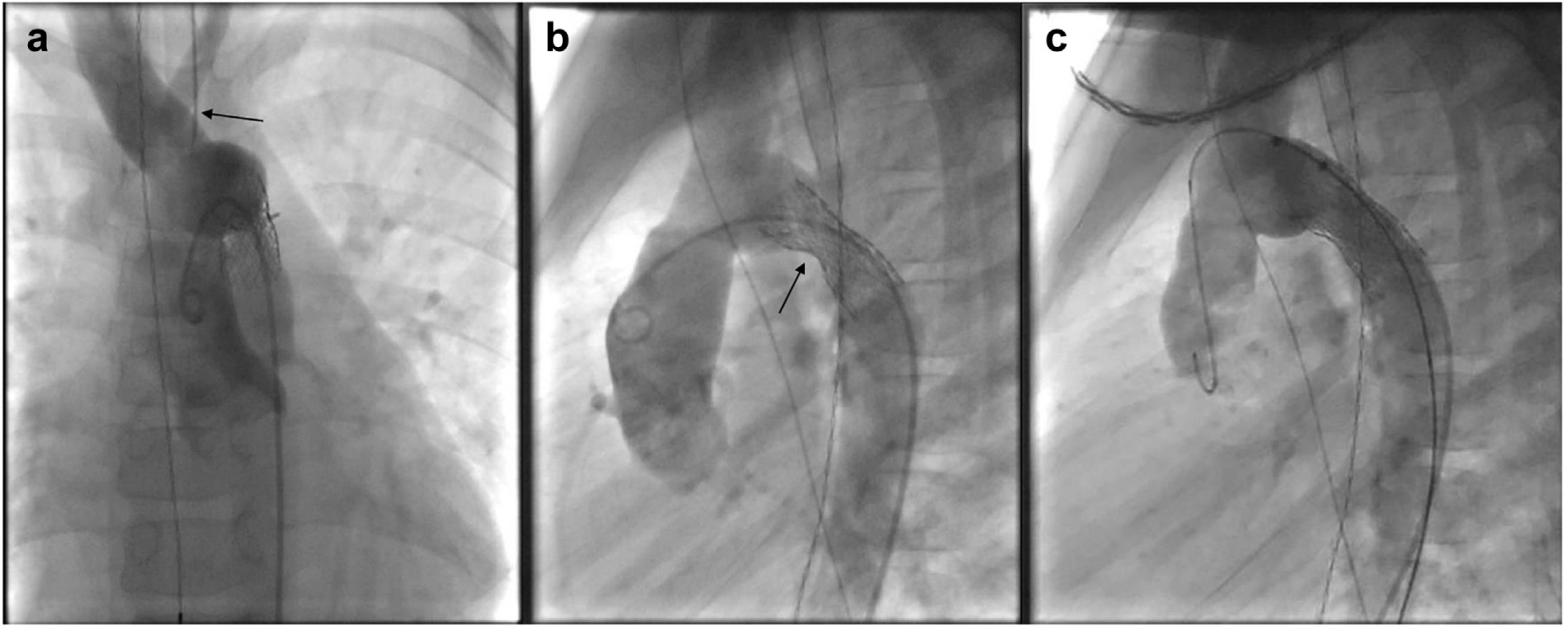
**Postoperative angiography.** (a) Follow-up angiography anterior posterior view demonstrating the carotid artery translocation (arrow). (b) Follow-up angiography lateral view demonstrating the implanted stent (arrow). (c) Final result after planed stent dilatation.

A follow-up echocardiography 2 ​weeks later and after 1 ​year showed a satisfying result without re-stenosis. Color Doppler imaging of both carotid arteries revealed regular flow on both sides. Angiography of the translocated carotid artery showed no stenosis (Figure 3a and b).

As planned, 1 ​year later, the implanted stent was dilated from 8 mm to 12 mm with a high-pressure balloon (Figure 3c).

## Discussion

RAAO occurs in up to 50% of patients with repaired IAA. The specific surgical method used for repair of IAA is found to be a risk factor for RAAO according to the study from LaPar and Baird.^3^

The current recommendation for treatment of RAAO is balloon angioplasty with or without stent placement.^3^, ^4^, ^5^ In our case, since the patient already had repeat balloon angioplasties, we preferred to proceed with a hybrid approach using stent implantation and carotid artery translocation due to the involvement of the offspring of LCCA. The advantage of this strategy was that there was no need for cardiopulmonary bypass nor profound hypothermia. Thus, known complications such as blood transfusions, hypoperfusion, prolonged intubation, or cerebrovascular events could be kept to a minimum. Furthermore, a more limited arch preparation with the hazard risk of recurrent laryngeal nerve laceration was possible.

We are convinced this hybrid management represents an interesting alternative approach for the treatment of complex aortic arch obstruction.

## Conclusion

To our knowledge, this is the first case described using an hybrid approach with carotid artery translocation and stenting of RAAO in a child. This could be considered as an alternative treatment of RAAO in children.

## Consent Statement

Patient consent was obtained for publication of this report and accompanying images.

## Funding

The authors have no funding to report.

## Disclosure statement

The authors report no conflict of interest.
